# 

**Published:** 1894-09-15

**Authors:** 


					fUE HOSPITAL. Supplied under Royal Warrant to Her Majesty the Queen. Sept. 15th, 1894.
fcbT 1 ? ? THE KING OF NATURAL TABLE WATER8. 1 ? M
dopMlte tH| dopMlia
.11 iii>
LKAun NORWICH HOSPl TAL
No. 416.-1 Vol. XVI.
Saturday,
Sept. 15th, 1894
. i_ni rmcmt PRICE TWOPENCE.
WITH FXTRA NUR8 NG SUPrLEWItNI. Per Annum, 108. 6d., post:tree
Wl I H CA I nH nunoiiiv ^ a Newspaper for Monthly Parts, 6d.; post free, 8d.
^e^^^miHsionliPti^'PBite^Kingd0Tn and Abroad-] Ditto per Annnm. 8s.6d., post free.

				

